# Bacterial Topography of the Healthy Human Lower Respiratory Tract

**DOI:** 10.1128/mBio.02287-16

**Published:** 2017-02-14

**Authors:** Robert P. Dickson, John R. Erb-Downward, Christine M. Freeman, Lisa McCloskey, Nicole R. Falkowski, Gary B. Huffnagle, Jeffrey L. Curtis

**Affiliations:** a Division of Pulmonary and Critical Care Medicine, Department of Internal Medicine, University of Michigan Health System, Ann Arbor, Michigan, USA; b Research Service, VA Ann Arbor Healthcare System, Ann Arbor, Michigan, USA; c Department of Microbiology & Immunology, University of Michigan Medical School, Ann Arbor, Michigan, USA; d Graduate Program in Immunology, Rackham Graduate School, University of Michigan, Ann Arbor, Michigan, USA; e Pulmonary and Critical Care Medicine Section, Medical Service, VA Ann Arbor Healthcare System, Ann Arbor, Michigan, USA; Icahn School of Medicine at Mount Sinai

## Abstract

Although culture-independent techniques have refuted lung sterility in health, controversy about contamination during bronchoscope passage through the upper respiratory tract (URT) has impeded research progress. We sought to establish whether bronchoscopic sampling accurately reflects the lung microbiome in health and to distinguish between two proposed routes of authentic microbial immigration, (i) dispersion along contiguous respiratory mucosa and (ii) subclinical microaspiration. During bronchoscopy of eight adult volunteers without lung disease, we performed seven protected specimen brushings (PSB) and bilateral bronchoalveolar lavages (BALs) per subject. We amplified, sequenced, and analyzed the bacterial 16S rRNA gene V4 regions by using the Illumina MiSeq platform. Rigorous attention was paid to eliminate potential sources of error or contamination, including a randomized processing order and the inclusion and analysis of exhaustive procedural and sequencing control specimens. Indices of mouth-lung immigration (mouth-lung community similarity, bacterial burden, and community richness) were all significantly greater in airway and alveolar specimens than in bronchoscope contamination control specimens, indicating minimal evidence of pharyngeal contamination. Ecological indices of mouth-lung immigration peaked at or near the carina, as predicted for a primary immigration route of microaspiration. Bacterial burden, diversity, and mouth-lung similarity were greater in BAL than PSB samples, reflecting differences in the sampled surface areas. (This study has been registered at ClinicalTrials.gov under registration no. NCT02392182.)

## IMPORTANCE

This study defines the bacterial topography of the healthy human respiratory tract and provides ecological evidence that bacteria enter the lungs in health primarily by microaspiration, with potential contribution in some subjects by direct dispersal along contiguous mucosa. By demonstrating that contamination contributes negligibly to microbial communities in bronchoscopically acquired specimens, we validate the use of bronchoscopy to investigate the lung microbiome.

## INTRODUCTION

The longstanding dogma that “the normal lung is free from bacteria” (1) has been overturned by the recent advent of culture-independent techniques of microbial identification. Results of such studies showed that healthy human lungs contain diverse bacterial communities (2–5). None of the >25 studies of healthy subjects that used molecular techniques to characterize bacteria in bronchoscopically obtained lung specimens has failed to detect bacteria (2–5). The bacterial load of bronchoscopically acquired specimens is roughly 100-fold greater than that of procedural control specimens (6–8). The viability of most of the bacteria recovered can be verified by using advanced cultivation techniques (9). In health, the lung microbial community composition determined by culture-independent techniques correlates with key features of host inflammation (7, 10), and its variation at spatially distinct lung sites within individuals is lower than intersubject community variation (2).

Culture-independent analyses of bronchoscopic specimens identify the oropharynx as the primary source for the bacterial lung microbiome in health (2, 7, 9). Bacterial communities detected in bronchoscopic specimens from healthy subjects more closely resemble those of the oropharynx than those of any competing source community (e.g., nasopharynx or inhaled air) (6, 9). Unlike in the gut, no novel bacteria have been identified within the lungs, and with rare exceptions (notably, *Tropheryma* [5, 11]), there is scant evidence of site-specific selective survival pressure or local reproduction during health (9). Thus, provided one accepts the validity of bronchoscopic sampling, these findings indicate that the healthy lung microbiome is determined largely by the balance between immigration of the oropharyngeal microbiota and its elimination by mucociliary clearance, coughing, and local host defenses (the adapted island model of lung biogeography) (2, 12–14).

However, with few exceptions (2, 7, 15, 16), studies of healthy individuals have been performed by using a single bronchoscopic specimen per subject and few have systematically analyzed the bacterial topography of the healthy human respiratory tract. Accordingly, there is uncertainty regarding the primary route of microbial immigration to the lungs, a crucial and currently unsettled issue that could involve direct dispersal of pharyngeal bacterial communities along the contiguous mucosa of the upper respiratory tract (URT) and lower respiratory tract (LRT) (4, 13) or subclinical aspiration of oropharyngeal contents (12, 13, 15, 17). Importantly, despite results congruent with those from bronchoscopy in a study using surgical lung specimens (18, 19), continued concern over potential contamination during the obligatory passage of the bronchoscope through the URT (15, 20) urges caution in interpreting results obtained by this technique.

To address these issues, we designed an experiment that depended, in part, on the use of protected specimen brushing (PSB), the gold-standard technique to avoid pharyngeal contamination in culture-dependent bronchoscopy studies (21–23), and in part on principles of microbial ecology that are well established to analyze the structure of bacterial communities in multiple environments (24).

## RESULTS

All of our study subjects (age range, 26 to 71 years) were HIV negative and without respiratory disease (Table 1). We analyzed samples independently of smoking status, which does not induce significant differences in the lung microbiome of otherwise healthy individuals (5). We obtained 17,392 ± 732 (mean ± standard deviation) sequence reads per specimen and did not exclude any specimens because of insufficient sequences.

**TABLE 1:** Characteristics of the eight subjects in this study

Characteristic	Value
Mean age (yr) ± SD	53 ± 15
No. (%) of females	5 (63)
No. of smokers (never/former/current)	6/2/0
Mean predicted FEV1%^a^ ± SD	92 ± 14
Mean predicted FVC%^b^ ± SD	90 ± 17

a FEV%, forced expiratory volume, % predicted.

b FVC%, forced vital capacity, % predicted.

**FIG 1:**
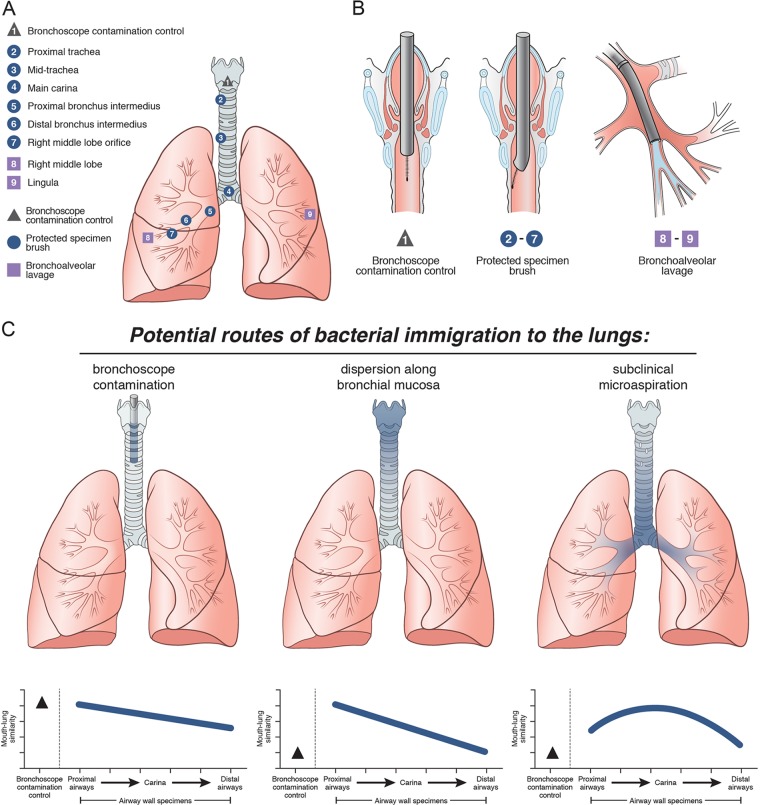
Experimental design and conceptual models. Eight subjects without respiratory disease underwent serial sampling of the LRT by bronchoscopy. (A) Sampling methods and locations. Numbers refer to the sampling order. (B) Schematic diagram of method: avoiding contact with airway mucosa for BCC (left), brushing a discrete area of airway mucosa with PSBs (middle), and sampling airways distal to the wedged bronchoscope by BAL (right). (C) Predicted bacterial topographic patterns for three possible routes of microbial immigration: bronchoscope contamination (indices of mouth-lung immigration peak with the BCC and decrease with serial sampling), dispersion along the bronchial mucosa (indices are low in BCC and high in proximal samples and decrease with distance from the pharyngeal source community), and microaspiration (indices are also low in BCC, peak at the main carina, and decrease with subsequent bronchial distance in upright subjects).

We systematically sampled the LRT microbiota by PSB of the airways and bronchoalveolar lavage (BAL) (Fig. 1A and B). With the subject supine under conscious sedation, the bronchoscope was advanced via the mouth through the vocal cords. A protected specimen brush was then extended from the protective sheath into the empty airway lumen without touching the airway wall; this specimen, designated the bronchoscope contamination control (BCC), represented only the microbiota introduced via contamination of the bronchoscope working channel during URT passage. We then performed six serial PSBs of the airway wall between the proximal trachea and the orifice to the right middle lobe (RML). Care was taken to sample the ventral surface of each airway site to minimize the contribution by secretions entering the LRT during the procedure. Finally, we performed BAL of the RML and lingula. The total bacterial DNA levels, as measured by quantitative PCR (qPCR) of the 16S rRNA gene, across the sampling sites and procedural and sequencing controls are shown in Fig. S1 in the supplemental material.

10.1128/mBio.02287-16.2 FIG S1  Quantification of bacterial DNA in control and biological specimens. The 16S rRNA gene was quantified by qPCR of airway brushings, BAL fluid, and all of the procedural and sequencing control specimens listed. Anatomic variation in bacterial density is shown in Fig. 3B. The data reported are mean values ± SEM. Download FIG S1, PDF file, 0.6 MB. Copyright © 2017 Dickson et al. 2017 Dickson et al. This content is distributed under the terms of the Creative Commons Attribution 4.0 International license.

This experimental design permits predictions of mouth-lung immigration that can be verified by the observed topographic pattern of LRT bacterial communities (Fig. 1C) (12, 13). If bacteria were primarily introduced simply because of contamination of the bronchoscope, ecological indices of mouth-lung immigration (mouth-lung community similarity, total bacterial burden, and community richness [12]) would be high in the BCC specimen but would then decay in subsequent airway samples because of dilution. Conversely, if the primary immigration route were dispersion along the bronchial mucosa, the signal in the BCC specimen would be minimal and there would be a distinct proximal-to-distal signal gradient. Finally, subclinical microaspiration would also show a minimal signal in the BCC specimen but the ecological signal of mouth-lung immigration would be highest near the carina because of the gravity dependence of the central airways in upright humans.

**FIG 2:**
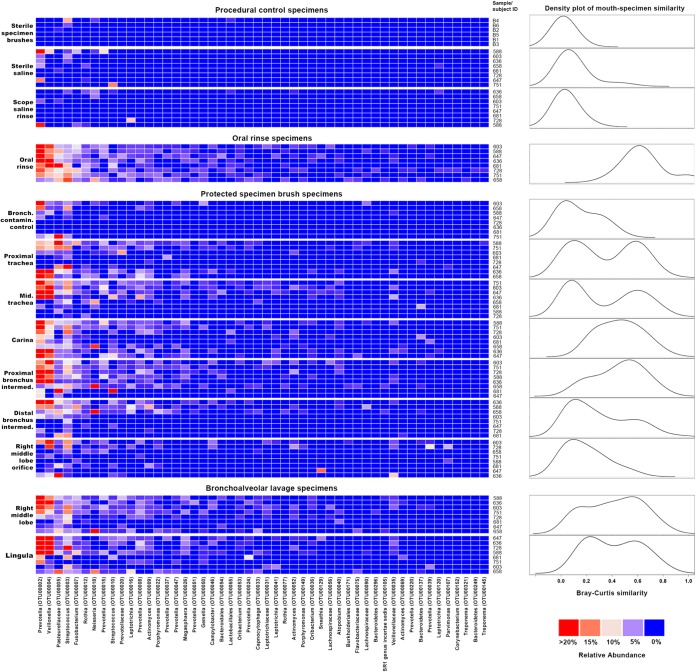
Bacterial taxa detected in airway, alveolar, and procedural control specimens. Taxa are listed in decreasing order of mean relative abundance in oral specimens. Red squares represent higher relative abundance (see color key at bottom right). On the right are plots of the kernel density estimates (bandwidth = 0.1) of Bray-Curtis similarity measurements comparing oral-to-specimen similarity (0, entirely different; 1, identical). The oral rinse plot reflects intragroup Bray-Curtis similarity. Plot heights are scaled to the relative density maximum.

Taxa detected in procedural control specimens, oral rinse specimens, and PSB and BAL specimens are presented in Fig. 2. The taxa detected in procedural control specimens bore little resemblance to prominent taxa in oral, airway, and alveolar specimens, though a single saline specimen from a single subject (no. 588) contained a *Prevotella* sp. (OTU00002) that was prominent in oral rinse, PSB, and BAL specimens across subjects. Oral rinse specimens were dominated by *Prevotella* (OTU00002), *Veillonella* (OTU00004), and *Streptococcus* (OTU00003), consistent with previous studies. Whereas these taxa were infrequently detected and in low abundance in BCC specimens, they were common and abundant in both PSB and BAL specimens. Density analysis (Fig. 2) showed that mouth-lung similarity was greatest across subjects at the carina and proximal bronchus intermedius, consistent with the predicted topography attributable to gravity-dependent microaspiration. Several subjects exhibited elevated mouth-lung similarity at the proximal and mid-trachea sites, consistent with direct dispersal along contiguous mucosa.

**FIG 3:**
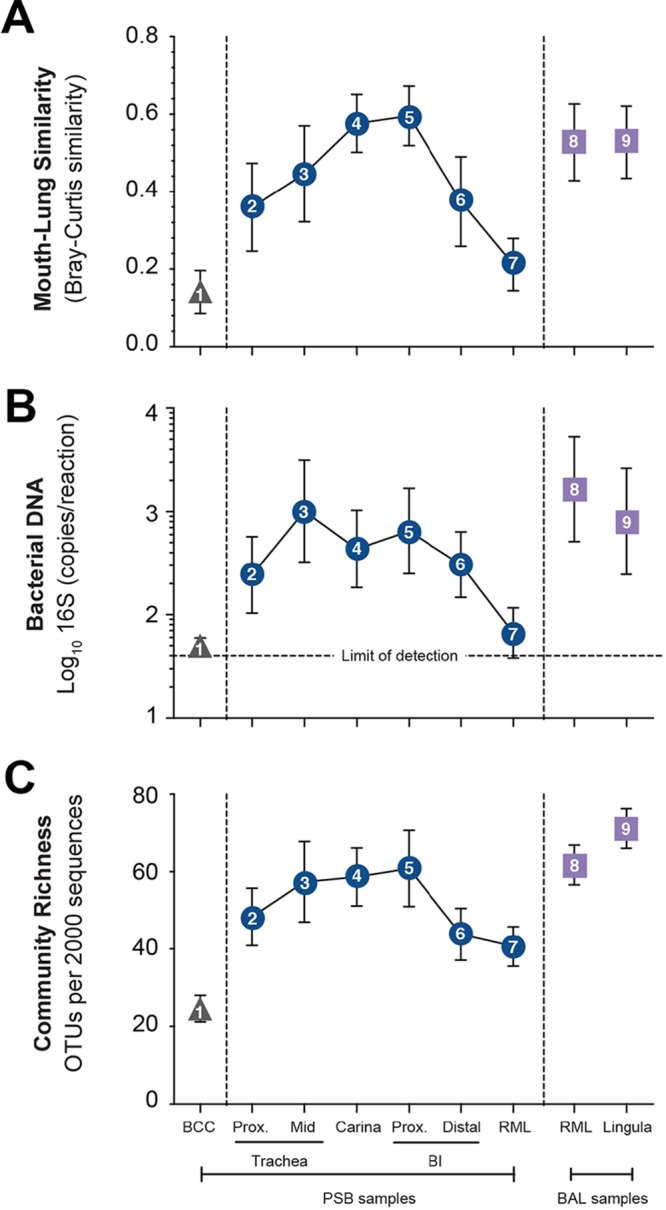
Bacterial topography of the healthy human LRT. Mouth-lung bacterial immigration along the LRT was quantified by mouth-lung community similarity (Bray-Curtis similarity) (A), bacterial DNA (log_10_ number of 16S copies per reaction determined by real-time qPCR) (B), and community richness (number of OTUs per 2,000 sequences) (C). Symbols are as in Fig. 1; Prox, proximal; BI, bronchus intermedius. By all indices, BCCs (triangle) exhibited less evidence of mouth-lung immigration than airway wall PSB specimens (squares) (*P* ≤ 0.001, paired Student *t* test) or BAL specimens (circles) (*P* ≤ 0.01, paired Student *t* test). Indices of mouth-lung immigration in airway PSB samples are nonlinear, consistent with the topographic pattern predicted in Fig. 1 for microaspiration in upright subjects. Data are mean values ± SEM (*n* = 8).

We then systematically compared indices of mouth-lung immigration at BCC, airway, and alveolar sites. We found that BCC specimens had minimal detectable signals and that by every ecological index of mouth-lung immigration (mouth specimen similarity, bacterial DNA, community richness), they were indistinguishable from the communities detected in preprocedure bronchoscope rinse specimens and sequencing reagent control specimens (*P* > 0.05 for all comparisons). Compared to airway PSB specimens from the same subjects, BCC specimens had significantly less mouth-lung community similarity (*P* = 0.0007) (Fig. 3A), bacterial DNA (*P* ≤ 0.0001) (Fig. 3B), and community richness (*P* ≤ 0.0001) (Fig. 3C). Similarly, compared to paired BAL specimens, BCC specimens had less mouth-lung community similarity (*P* = 0.0002) (Fig. 3A), bacterial DNA (*P* = 0.0016) (Fig. 3B), and community richness (*P* ≤ 0.0001) (Fig. 3C). Because BAL samples were collected last, if bronchoscope contamination were a significant factor, they would have had the lowest community richness and least similarity to the mouth community, which was not the case.

Moreover, sequences detected in procedural and reagent control specimens were significantly distinct from sequences detected in PSB and BAL specimens (*P* ≤ 0.05 for all comparisons). The three most abundant taxa detected in sequences from control protected specimen brushes (brushes that were handled aseptically without use in subjects and then processed in parallel with other specimens) were classified as *Ruminococcus* sp. (OTU00062), *Pseudomonas* sp. (OTU00006), and *Acinetobacter* sp. (OTU00066), comprising 29% ± 11% (mean ± the standard error of the mean [SEM]) of all PSB control sequences. In contrast, these three operational taxonomic units (OTUs) collectively made up only 2% ± 1% of the sequences from airway wall specimens. The three most abundant taxa detected in sequences from unused sterile saline were classified as *Pseudomonas* sp. (OTU00006), *Prevotella* sp. (OTU00002), and *Yersinia* sp. (OTU00044), comprising 27.51% ± 8.33% of all such sequences. Collectively, these three OTUs made up 19.60% ± 3.04% of the sequences from oral rinse specimens and 14.78% ± 2.84% of the sequences from BAL specimens. The overlap between saline and BAL specimens was attributable to a single specimen of sterile saline (no. 588) that had a high abundance of a single OTU (*Prevotella* sp. OTU00002) (Fig. 2). Additionally, when collective community structures were examined by using permutational multivariate analysis of variance (PERMANOVA; Adonis), BCC specimen communities were again indistinguishable from communities of reagent control specimens (*P* > 0.05) but differed significantly from the communities of paired airway PSB and BAL specimens (*P* < 0.05 for both).

Thus, we conclude that contamination during URT passage has a minimal effect on bronchoscopically acquired respiratory specimens. This finding is consistent with our previous observation that BAL fluid communities are not appreciably altered by the passage of a bronchoscope through the nasopharynx versus the oropharynx, despite the starkly dissimilar communities present at those URT sites (8, 13).

We next studied the bacterial topographic data to determine the relative contributions of mucosal dispersion and microaspiration to the microbial immigration to healthy human lungs. None of the indices of mouth-lung immigration were greatest in the proximal trachea (Fig. 3), as would be predicted for dispersion along contiguous bronchial mucosa surfaces (Fig. 1C). Rather, indices of mouth-lung immigration in PSB samples peaked between the proximal trachea and the carina and subsequently decreased with greater distance along the airways. This ecological trend could be seen most distinctly in mouth-lung similarity (Bray-Curtis distance) (Fig. 3A), which failed to fit the linear relationship predicted for dispersion along the bronchial mucosa (*P* > 0.05) but did fit the parabolic (quadratic) relationship predicted for microaspiration (*P* = 0.0017), which resulted in less loss of information by the Akaike information criterion (AIC; linear [23.279] versus quadratic [14.847] models). While some subjects exhibited evidence of bacterial immigration at the proximal and mid-trachea sites (see the bimodal density plots for these sites in Fig. 2), the strongest and most uniform evidence of mouth-lung immigration was found at the carina and bronchus intermedius (see the unimodal distribution in Fig. 2). Taken together, the bacterial topography data from PSB samples were most consistent with microaspiration being the primary immigration route (Fig. 1C), though concurrent immigration from mucosal dispersion (Fig. 1B) cannot be excluded and likely contributes in some healthy subjects.

Comparison of the communities detected by the two sampling modalities shows that BAL specimens exhibited greater signals of mouth-lung immigration than did distal PSB specimens, independent of the measurement used (*P* ≤ 0.05 for all three measurements) (Fig. 3). We interpret these results to reflect differences in the surface areas sampled; whereas PSB samples approximately 1 cm^2^ of the airway mucosa, BAL fluid of a wedged subsegment samples approximately 1/40 of the total surface area of the lungs (25) or approximately 17,500 cm^2^. Hence, even though the mucosal density of the lung microbiota decreased with distance from the central airways, the larger surface area sampled by BAL permits the detection of a greater bacterial signal, with minimal influence from bronchoscopic contamination. The bacterial burdens we detected via PSB and BAL were comparable to values reported in prior studies (4, 6).

**FIG 4:**
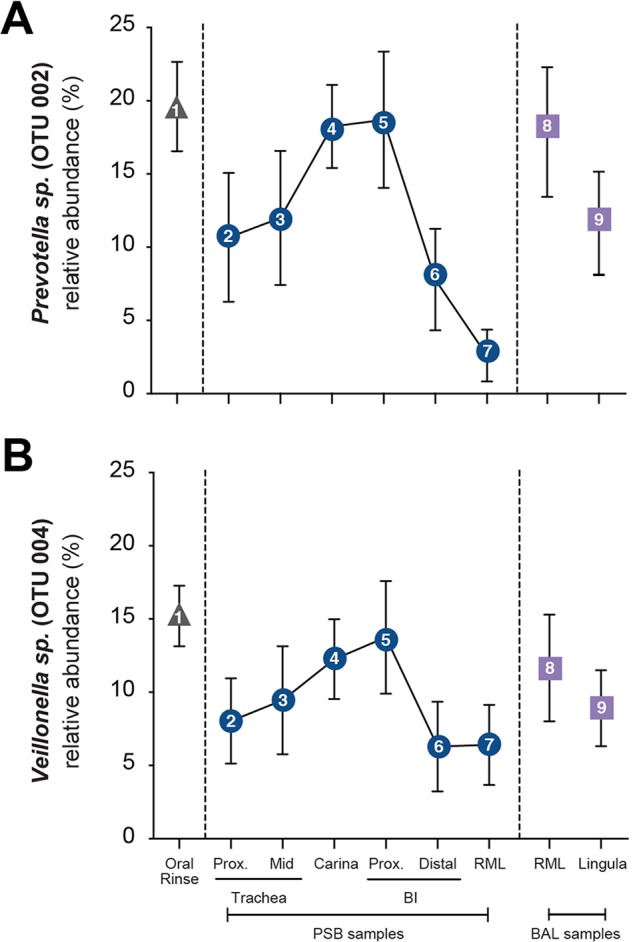
Bacterial community membership along the healthy human LRT. The relative abundances of the two most abundant bacterial community members in oral rinse specimens, *Prevotella* sp. (OTU002) (A) and *Veillonella* sp. (OTU004) (B), among LRT communities at various locations are graphed. Symbols are as in Fig. 1. Note that the leftmost sample is oral rinse rather than BCC as in Fig. 1C and 2. The relative abundances of both OTUs in LRT PSB samples are nonlinear, peaking at the carina and proximal (Prox.) bronchus intermedius (BI), consistent with the predicted pattern of microaspiration in upright subjects. Data are mean values ± SEM (*n* = 8).

Bacterial community membership detected in airway communities mirrored the pattern identified in indices of mouth-lung immigration (Fig. 4). The relative abundances along the LRT of the two most abundant bacterial community members detected in oral specimens, *Prevotella* sp. (OTU002) (Fig. 4A) and *Veillonella* sp. (OTU004) (Fig. 4B), each peaked at the carina and proximal bronchus intermedius, with significantly smaller fractions at both more proximal and more distal airway sites (*P* ≤ 0.01). The topographic distribution of these community members’ relative abundance was also most consistent with the predicted pattern of microaspiration (Fig. 1C).

## DISCUSSION

Results of this systematic ecological survey of the healthy human LRT demonstrate three major points. First, when performed by an experienced bronchoscopist, with care to avoid gravity-dependent proximal tracheobronchial surfaces, contamination during URT passage has a minimal effect on bronchoscopically acquired respiratory specimens. Second, when combined with similar care to minimize errors in subsequent laboratory and bioinformatic analyses (especially through the extensive use of environmental controls), bronchoscopic sampling and next-generation sequencing can reliably define the membership of LRT bacterial communities. Third, our data favor microaspiration as the primary source of bacterial immigration to human lungs in health, although we cannot exclude a contribution from contiguous mucosal dispersion. These findings, and especially these methodological considerations, provide crucial reference information to define how bacteria contribute to infectious and noninfectious lung diseases, which collectively cause ~15% of worldwide deaths.

Verifying that bronchoscopic sampling can be used reliably to study the human lung microbiome has several important implications. Although an invasive procedure, outpatient investigative bronchoscopy under moderate conscious sedation can be performed safely not only on healthy volunteers but also on those with established lung diseases (26, 27). Investigative bronchoscopy is increasingly being incorporated into multicenter observational trials to link microbiome analyses to intermediate biomarkers of disease activity and progression (28–30). The current results should dispel concerns that the substantial investment of these undertakings is misguided. Bronchoscopy is also well suited to the careful topographical analysis needed to define how the composition of lung bacterial communities responds to the markedly disparate gradients of temperature, oxygen tension, and other variables along the human LRT. Because lung bacterial communities show pronounced anatomic heterogeneity in advanced chronic obstructive pulmonary disease (31) and likely will in other chronic pulmonary diseases, this consideration will take on special significance in the attempt to define how interactions between hosts and microbes contribute to the pathogenesis of different airway diseases. Additionally, our results provide support for the complementary capacities of PSB and BAL to sample the conducting airways and distal lungs, respectively, another important distinction in chronic lung diseases. Further, being able to rely on bronchoscopic sampling as a standard is an essential first step toward testing the validity of less invasive techniques, such as analysis of sputum or exhaled breath condensate, which pose even larger issues of potential contamination when paired with highly sensitive culture-independent microbiological techniques. Hence, bronchoscopic analysis of human LRT microbial communities has the potential to advance pulmonary research in multiple ways, not the least by informing strategies to modify lung bacterial communities therapeutically.

In principle, bronchoscopically acquired lung specimens are vulnerable to contamination from two major sources, the URT (via passage of the bronchoscope through the pharynx [16]) and the bacterial DNA present in laboratory reagents (32). Our findings clarify the relative contributions of both sources and demonstrate that the concern for URT contamination is largely unfounded. In contrast, reagent contamination is a real, underappreciated problem that must be addressed via systematic sequencing of “negative” procedural control specimens (32). Fortunately, with this precaution, the source of bacterial DNA becomes clear.

Consistent with numerous previous studies of the healthy human bacterial lung microbiome (2, 4, 5, 7, 16), we found that airway and lung communities resemble oropharyngeal communities, with minimal evidence of site-specific enrichment by reproducing bacteria. We did not identify lung-specific taxa distinct from oropharyngeal taxa or the stochastic background taxa detected in procedural and sequencing control specimens. Our experimental design, with its meticulous collection of control specimens (including a dedicated postlaryngeal lumenal brush), demonstrated that the microbial signal detected in airway and alveolar specimens is not an artifact of pharyngeal contamination. Instead, this similarity of the mouth and lung microbiotas is far more plausibly explained by the ecological contiguity of these two anatomic compartments and by the ubiquity of subclinical microaspiration (as has been demonstrated repeatedly by using radiographic techniques [17, 33, 34]). In contrast, all negative procedural control specimens (including sterile saline, laboratory reagents, and unused specimen brushes) contained evidence of bacterial DNA when sequenced. The bacterial signal introduced by these sources—referred to variously as the “kitome” and “contaminome”—is unavoidable in low-biomass microbiome studies (7, 32). Though such a spurious signal cannot be excluded from studies such as ours, it can be managed if prospectively sought and properly analyzed. We have adopted and strongly support recommendations on minimizing the risk of systematic bias and false grouping in low-biomass microbiome studies (32), i.e., sequencing of multiple “negative” controls for all potential sources of contamination, using a single DNA extraction kit for all specimens, randomizing the order in which specimens are processed, and systematically comparing the taxa detected in negative controls with those in biological specimens to determine the relative influence of reagent contamination.

Our results provide further support for the “adapted island model” (2, 3, 12), in which the lung microbiome in health is determined by the balance of microbial immigration (here identified as chiefly due to microaspiration) and elimination (6, 12, 13), with a minimal detectable influence from selective pressure on reproducing communities (9). Our findings agree with imaging studies indicating that subclinical aspiration is common in healthy subjects (17, 33, 34). Further studies are needed to elucidate how specific pulmonary and extrapulmonary diseases (13) alter this balance of ecological forces and, conversely, how deviations in the structure of bacterial respiratory tract communities from the neutral, orally derived microbiome participate in the pathogenesis of acute and chronic lung diseases. Given the meteoric rise in the worldwide prevalence of lung diseases, especially asthma and chronic obstructive pulmonary disease, these are important goals.

## MATERIALS AND METHODS

### Participants.

We conducted all of our investigations according to principles of the Declaration of Helsinki. The protocol was approved by the Human Subject Subcommittee of the VA Ann Arbor Health Care System. Participants were a subset of healthy volunteers recruited in the Lung HIV Microbiome Project (ClinicalTrials.gov registration no. NCT02392182).

For additional details of all aspects of the methods used in this study, see Text S1 in the supplemental material.

10.1128/mBio.02287-16.1 TEXT S1  Supplemental methods used in this study. Download TEXT S1, DOC file, 0.3 MB. Copyright © 2017 Dickson et al. 2017 Dickson et al. This content is distributed under the terms of the Creative Commons Attribution 4.0 International license.

### Sample acquisition and processing.

Oropharyngeal microbiotas were sampled by using an oral rinse collected before local anesthesia. We have previously published our bronchoscopic technique (2, 5), although this study omitted gastric sampling and added PSBs. Before each procedure, a control saline sample was collected by aspiration through the bronchoscope. After administration of lidocaine to the URT and sedation, the bronchoscope was inserted through the mouth and advanced quickly and without suctioning to the vocal cords. The sequence of sampling is detailed in Results and Fig. 1. BAL fluid was processed as previously described (8, 35).

We collected reagent water controls at the time of DNA isolation and processed them in parallel with study specimens.

### Bacterial DNA isolation.

We identified bacteria by sequencing bacterial 16S rRNA genes by using previously described methods of genomic DNA extraction and amplification (2, 8), V4 region amplification with previously published primers (36), a dual-indexing sequencing strategy (37), and sequencing with the Illumina MiSeq platform. Quantification of the bacterial 16S rRNA gene was performed by real-time PCR as previously described (8).

### 16S DNA sequencing and statistical analysis.

We processed sequence data by using mothur v.1.33.0 (38) at a minimum sequence length of 250 bp (39). We generated a shared community file and a genus level grouping file by using OTUs binned at 97% identity generated in mothur. OTU classification was performed by using the mothur implementation of RDP Classifier (40) and its taxonomy training set 9. OTUs were numbered by mothur on the basis of their relative frequencies in the entire analysis.

We performed microbial ecology analysis with the vegan package 2.0-4 and mvabund in R (41–43). For relative abundance analysis, samples were normalized to the percentage of total reads, and then we restricted the analysis to OTUs present at >1% of the sample population; for diversity analysis, all OTUs were included. We determined the significance of differences in community composition by using PERMANOVA (Adonis) with 1,000 permutations and constructed both linear and quadratic mixed models by using the lmer function in the R packages lme4 and lmerTest. We compared the relative qualities of fit of these two models to our data by using the AIC, which defines the trade-off among models between goodness of fit and complexity as the relative loss of information (44). Heat maps were generated in R with the ComplexHeatmap package (45), splitting groups by specimen type and with cluster_columns=F so as to maintain the rank order of the oral cavity-associated taxa. Density plots were generated on the basis of a Bray-Curtis similarity measurement by using the density function from the R Stats package. All statistical analyses were performed in R and GraphPad Prism 6. We compared means via paired *t* test and paired ANOVA with Tukey’s multiple-comparison *post hoc* test, as appropriate. Investigators were not blinded to specimen sources during analysis.

### Identification of procedural contaminants.

To identify potential sources of contamination in sequencing, we collected multiple procedural controls, including saline used in bronchoscopy, sterile water used in library preparation, unused PSBs, and AE buffer used in DNA isolation. These procedural controls and mock community standards, containing known ratios of preidentified bacterial DNA, were analyzed in the same sequencing run as the study specimens. To minimize false pattern formation due to reagent contamination (32), we processed specimens in a randomized order.

### Accession number(s).

The bacterial sequence data obtained in this study are available via the NCBI Sequence Read Archive (GenBank accession no. SRP072219).
