# Supplementary material for: Bacterial Topography of the Healthy Human Lower Respiratory Tract
Source: mBio. 2017 Feb 14;8(1):e02287-16. doi: 10.1128/mBio.02287-16 (PMC5312084; doi:10.1128/mBio.02287-16)
Supplement: FIG S1 [file mbo001173194sf1.pdf]

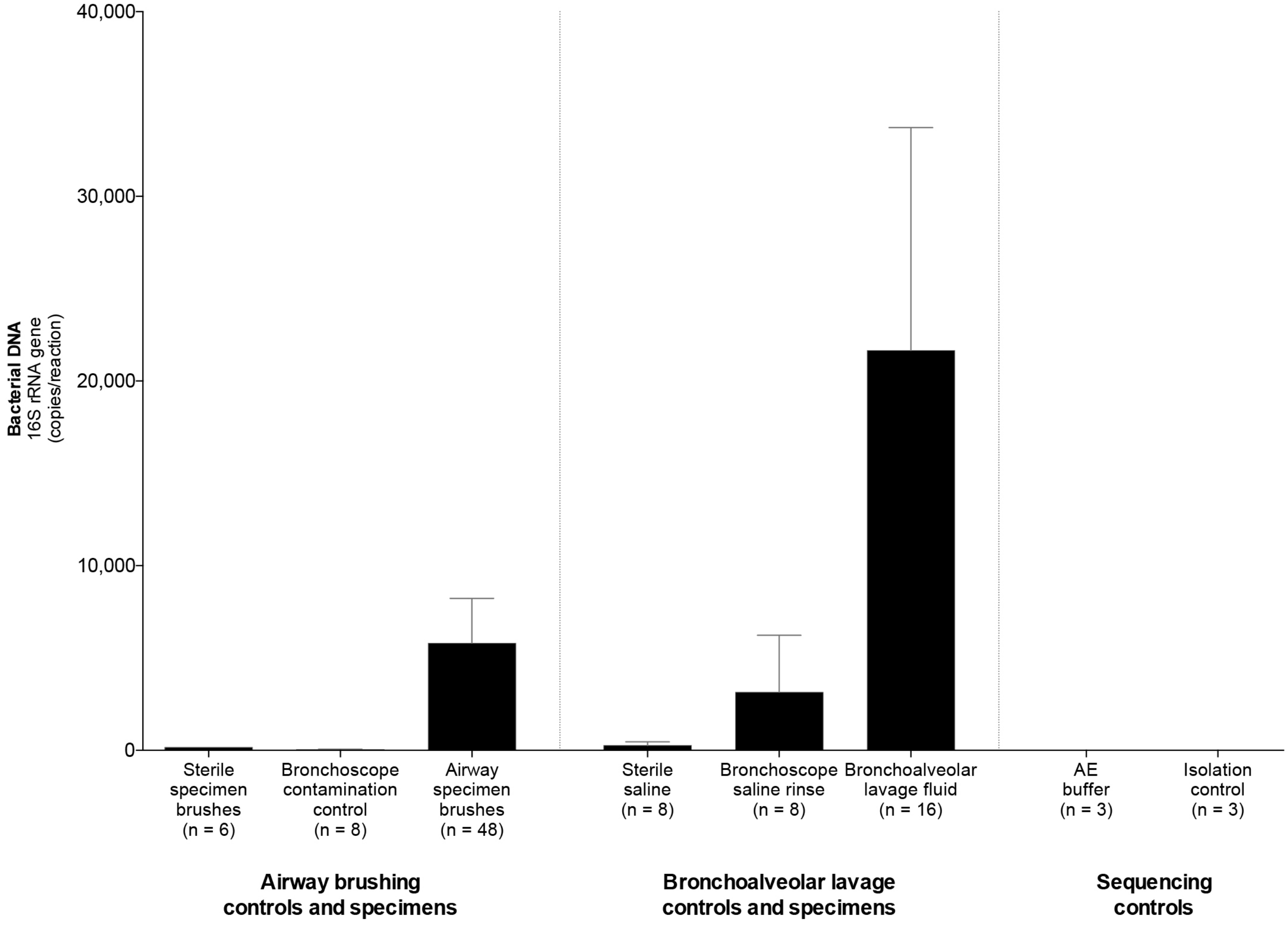

Supplemental FIG 1. Quantification of bacterial DNA in control and biologic specimens. The 16S rRNA gene was quantified using qPCR of airway brushings, bronchoalveolar lavage fluid, and all listed procedural and sequencing control specimens. Anatomic variation in bacterial density is shown in Figure 3B. Values reported as mean ± SEM.
