# Supplementary material for: Bacterial Topography of the Healthy Human Lower Respiratory Tract
Source: mBio. 2017 Feb 14;8(1):e02287-16. doi: 10.1128/mBio.02287-16 (PMC5312084; doi:10.1128/mBio.02287-16)
Supplement: TEXT S1 [file mbo001173194s1.doc]

**online data supplement**

**The bacterial topography of the healthy human
lower respiratory tract**

Robert P. Dickson ^1^*, John R. Erb-Downward ^1^*, Christine M. Freeman ^1,2^, Lisa McCloskey ^1^, Nicole R. Falkowski ^1^, Gary B. Huffnagle ^1,3,4^, Jeffrey L. Curtis ^1,4,5^

# Methods

## Ethics statement.

We conducted all clinical investigations according to the principles of the Declaration of Helsinki. The study protocol was approved by the Human Subject Subcommittee of the VA Ann Arbor Healthcare System (FWA 00000348), where all interactions with research participants occurred. All participants understood the study’s purpose and provided written informed consent before any research procedures.

## Participants.

The eight participants were a subset of volunteers recruited in the Lung HIV Microbiome Project from the southeast Michigan community, primarily by means of the University of Michigan clinical trials website. All participants underwent a complete history and physical examination by a Pulmonologist, pulmonary function testing, chest imaging, prospective collection of medication history, and complete blood count with differential, coagulation studies and comprehensive chemistry panel. The study was registered with ClinicalTrials.gov (<https://clinicaltrials.gov/> NCT02392182).

Inclusion criteria were men and women aged 18 to 80 years. Exclusion criteria were known history of pulmonary disease, reported fever, cough, or upper respiratory symptoms in the previous four weeks, or use of antibiotics or immunosuppressive medications in the past three or six months, respectively.

## Sample acquisition and processing.

We have previously published our research bronchoscopic technique , although for this study, the protocol and informed consent documents were amended to omit gastric sampling and to add the protected specimen brushings (PSB). Before each procedure, a control sample of freshly-opened saline was collected by aspirating through the bronchoscope, then this saline was used for that procedure.

Bronchoscopy was performed with the participant in the recumbent position in a dedicated suite with negative pressure ventilation, using standard clinical universal precautions including surgical masks for all personnel. We first administered local anesthetic to the upper respiratory tract (4% lidocaine administered by nebulizer and by spraying the posterior pharynx before sedation). After moderate conscious sedation, using diphenhydramine pre-medication, then fentanyl and midazolam, all given intravenously, the bronchoscope was inserted through the mouth and advanced quickly and without suctioning to the vocal cords. Lidocaine 4% was gently administered as 1 mL x 4 aliquots directly on the vocal cords.

The sequence of sampling, including bronchoscope-contamination controls, airway PSBs, and bronchoalveolar lavage (BAL) is detailed in the Results section. Saline for BAL was pre-warmed to 37°C to minimize coughing. In addition to PSB #1, which was deployed in the airways but which did not contact the airway wall, we included mock PSBs (brushes that were handled aseptically without use in subjects, then processed in parallel with other specimens) in all sequence runs. To minimize the chance of bacterial replication outside the subject, we held the recovered BAL fluid samples, which were recovered by gentle manual suction, and PSB specimens on ice until the time of processing. We collected reagent water controls at the time of DNA isolation and processed them in parallel with study specimens.

## Bacterial DNA isolation.

We identified bacterial community members using sequencing of the bacterial 16S rRNA gene, a small and highly-conserved locus of the bacterial genome that permits genus- and species-level identification. Our methods of genomic DNA extraction and touchdown PCR amplification have been described . We amplified the V4 region of the 16s rRNA gene from each sample using published primers [^4^](#_ENREF_4) and the dual-indexing sequencing strategy developed by the laboratory of Patrick D. Schloss [^5^](#_ENREF_5). We used Accuprime High Fidelity Taq instead of Accuprime Pfx SuperMix. Primary PCR cycling conditions were 95°C for two minutes, followed by 20 cycles of touchdown PCR (95°C 20 seconds, 60°C 20 seconds and decreasing 0.3 degrees each cycle, 72°C 5 minutes), then 20 cycles of standard PCR (95°C for 20 seconds, 55°C for 15 seconds, and 72°C for 5 minutes), and finished with 72°C for 10 minutes.

We performed sequencing using the Illumina MiSeq platform, using a MiSeq Reagent Kit V2 (500 cycles), according to the manufacturer’s instructions with modifications found in the Schloss Standard Operating Procedure [^6^](#_ENREF_6).

## 16S DNA sequencing and statistical analysis.

We processed sequence data using the software mothur v.1.33.0 according to the Standard Operating Procedure for MiSeq sequence data [^6^](#_ENREF_6) using a minimum sequence length of 250 basepairs [^7^](#_ENREF_7). Details regarding the pipeline have been previously published[^5^](#_ENREF_5). We generated a shared community file and a phylotyped (genus-level grouping) file using operational taxonomic units (OTUs) binned at 97% identity generated using the dist.seqs, cluster, make.shared and classify.otu commands in mothur. Classification of OTUs was carried out using the mothur implementation of the Ribosomal Database Project (RDP) Classifier[^8^](#_ENREF_8) and the RDP taxonomy training set 9 (fasta reference = trainset9_032012.pds.fasta, taxonomy reference = trainset9_032012.pds.tax), available on the mothur website (<http://www.mothur.org/wiki/RDP_reference_files>).

OTUs were numbered by mothur based on their relative frequency among the entire analysis. Bacterial sequence data from this study are available via the NCBI Sequence Read Archive (<http://www.ncbi.nlm.nih.gov/sra>) (accession number SRP072219).

We performed microbial ecology analysis using the *vegan* package 2.0-4 and *mvabund* in R[^9-11^](#_ENREF_9). For relative abundance analysis, samples were normalized to the percent of total reads, then we restricted analysis to OTUs present at greater than 1% of the sample population (453 OTUs); for diversity analysis, all OTUs were included. Rarefaction was performed using *vegan*’s *rarefy* function with default parameters to 2000 sequences per specimen, single iteration. We determined significance of differences in community composition using PERMANOVA (*adonis*) with 1000 permutations. Bray-Curtis distance was calculated based upon a normalized OTU table. Linear Mixed Models were constructed as (Y~X+(1|subjectID)) for the linear model or (Y~poly(X,2)+(1|subjectID)) for the quadratic model using the lmer function in the R-packages lme4 and lmerTest. Significance testing of the goodness of fit of the linear and quadratic model was determined using ANOVA on the model generated by the lmer function. We compared the relative quality of fit of these two models to our data using Akaike Information Criterion, which defines the trade-off amongst models between goodness of fit versus complexity, as the relative loss of information[^12^](#_ENREF_12). All analyses were performed in R and GraphPad Prism 6. We compared means via paired t-test and paired ANOVA with Tukey’s multiple comparisons post hoc test as appropriate. Investigators were not blinded to intervention status of specimens during analysis.

## Identification of procedural contaminants.

Reagents used in DNA isolation and library preparation contain bacterial DNA that can contaminate sequence-based studies of microbial communities [^13^](#_ENREF_13). To identify potential sources of contamination in sequencing, we collected multiple procedural controls, including saline used in bronchoscopy, sterile water used in library preparation, unused PSBs, and AE buffer used in DNA isolation. These procedural controls and mock community standards were analyzed as quality controls in each sequencing run. To minimize the risk of false pattern formation due to reagent contamination [^13^](#_ENREF_13), we processed specimens in a randomized order.

# references
